# Aboriginal and Torres Strait Islander maternal and child health and wellbeing: a systematic search of programs and services in Australian primary health care settings

**DOI:** 10.1186/1471-2393-14-251

**Published:** 2014-07-30

**Authors:** Crystal Jongen, Janya McCalman, Roxanne Bainbridge, Komla Tsey

**Affiliations:** The Cairns Institute, James Cook University, PO Box 6811, Cairns, QLD 4870 Australia

**Keywords:** Antenatal, Postnatal, Indigenous Australians, Pregnancy, Women’s health

## Abstract

**Background:**

Persistent disparities in pregnancy and birth outcomes between Aboriginal and Torres Strait Islander and other Australians evidence a need to prioritise responsive practice in Maternal Child Health (MCH). This study reviewed the existing knowledge output on Aboriginal and Torres Strait Islander MCH programs and services with the objective to advance understanding of the current evidence base and inform MCH service development, including the identification of new research priorities.

**Methods:**

A systematic search of the electronic databases Informit, Proquest, PubMed, Scopus, Wiley, and Cinahl, and 9 relevant websites was undertaken for the period 1993–2012. The reference lists of MCH program reviews were hand-searched for additional relevant studies which met the eligibility criteria. The study designs of included publications were classified and the characteristics extracted and categorized. Evaluation quality was assessed using the Effective Public Health Practice Project (EPHPP) Quality Assessment Tool for Quantitative Studies and the Critical Appraisal Skills Program (CASP) tool for qualitative studies.

**Results:**

Twenty-three search results were identified for inclusion, with the majority published in 2003–2012. Fifty two percent of publications reported on programs and services operating out of Aboriginal Community Controlled Health Organisations, with antenatal and postnatal care the main intervention type/s, and health promotion/education and advice/support the most common intervention component. Outcomes such as increased antenatal attendance and higher infant birth weights were reported in some intervention studies, however methodological quality varied considerably with quantitative studies typically rated weak.

**Conclusion:**

The prevalence of community controlled and/or community-based programs is significant given the health and wellbeing implications of self-determination. While the literature highlights the promise of many intervention models and program components used there are some significant gaps in the documentation and implementation of important MCH interventions. Similarly, while positive health outcomes were reported there are issues with key measures used and study quality. This review highlights the need to improve the quality of evaluations of MCH programs for Aboriginal and Torres Strait Islander women and to address the key evidence gaps in responding to their health and wellbeing needs.

## Background

Early life experiences, beginning with those of the developing foetus, play an important role in creating the foundations for health and wellbeing throughout the lifetime [1]. Due to the impact of Maternal and Child Health (MCH) on general population health, enhancing MCH is a key global health issue and a significant focus of worldwide public health strategies [2]. While improvements have been made in health outcomes for mothers and infants globally, Indigenous people worldwide still experience much poorer MCH outcomes compared to non-indigenous populations [3]. The significant gaps in health and wellbeing equity between Indigenous and non-Indigenous populations in Australia, as in other settler colonial countries, have been well described; as has been evidence of achievement of health targets which has shown that it is possible to improve health [4].

There is a lack of a quality evidence base to guide Indigenous health and wellbeing programs globally, and particularly of intervention research focused on testing and analysing the effectiveness of potential solutions [5, 6]. In contrast, reviews of Aboriginal and Torres Strait Islander MCH in Australia have identified literature documenting MCH responses and interventions. However, they also identified important gaps in the evidence base guiding Aboriginal and Torres Strait Islander primary health care MCH strategies. Prior to embarking on new initiatives in primary health care research or practice, it is important to assess the current state of evidence.

Two prominent early reviews of the literature provide a range of evidence on MCH issues and responses for Aboriginal and Torres Strait Islander women and babies [7, 8]. Assessing studies of standout Aboriginal and Torres Strait Islander MCH programs, these reviews reported on outcomes identified in the literature, such as improvements in antenatal attendance, decreased pre-term births and improvements in infant birth weight, associated with MCH programs. Eades [8] provided information on factors affecting key poor birth outcomes most likely to be associated with primary health care, including genital infections, urinary tract infections (UTI’s), sexually transmitted infections (STI’s) and tobacco and alcohol consumption during pregnancy. Herceg [7] also identified a number of common factors present in successful Aboriginal and Torres Strait Islander MCH programs, including programs being community-based and/or community controlled, the presence of Aboriginal and Torres Strait Islander and female staff, outreach, home-visiting and transport.

Importantly, the review by Herceg [7] identified evidence gaps for key MCH issues such as tobacco, alcohol and other drug use in pregnancy and effective health promotion interventions. This review highlighted the lack of quality evidence for interventions, and stressed the need for high quality evaluations of programs. A later review extended this assessment in a review of evaluations of antenatal care programs for Aboriginal and Torres Strait Islander women [9]. This review found particular weakness in the diversity of evaluation designs and the quality of reported data in studies assessed. The authors reiterated the need to collect and report good quality longitudinal data about care programs to demonstrate clinically relevant differences in perinatal outcomes.

This systematic search was developed in response to the Queensland Government Centre for Social Science Innovation (QCSSI) research priority area of Aboriginal and Torres Strait Islander MCH. This review provides an overview of the literature describing or evaluating Aboriginal and Torres Strait Islander MCH programs and services in Australian primary health care settings from 1993–2012. Primary health care responses included in this review are first level health care services providing antenatal and postnatal care, and maternal and child care. These primary care responses, operating out of mainstream community health services, government health services and Aboriginal Community Controlled Health Services, are increasingly being recognised as the preferred approach for effecting key MCH outcomes, such as reducing the prevalence of low infant birth weight and pre-term births [8]. The review aims to: 1) identify the number of publications on MCH programs and services; 2) describe their main characteristics; 3) outline the reported outcomes; and 4) assess the methodological quality of intervention studies. The objective of this review is to advance understanding of the current evidence base guiding Aboriginal and Torres Strait Islander MCH and wellbeing practices and to informprimary health care MCH service development, including the identification of new research priorities.

## Methods

### Search strategy

**Figure 1:**
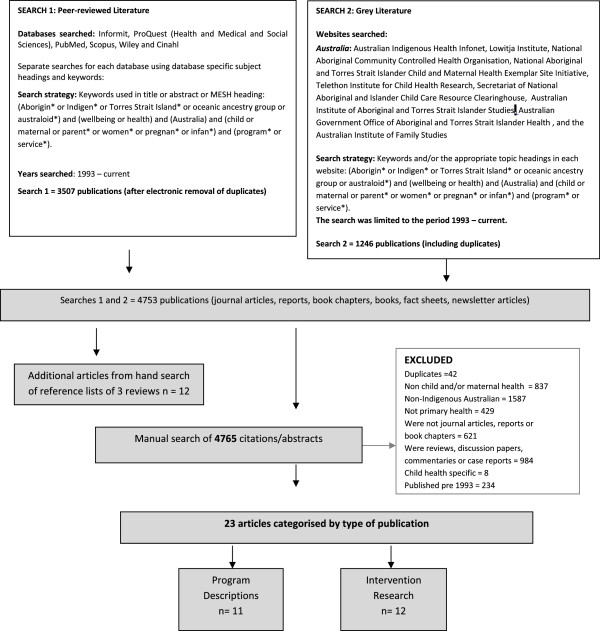
**Flowchart of search strategy.**

Figure 1 summarises the databases searched, the search terms used, the exclusion criteria, and classification of studies. Consistent with methods detailed in Cochrane guidelines for systematic reviews [10] and those used in previous systematic reviews [11, 12], the search strategy comprised three steps. First, consultation with a qualified librarian identified six electronic databases: Informit, Proquest (Health and Medical and Social Sciences), PubMed, Scopus, Wiley, and Cinahl. The following terms were searched in either the title or abstract, article or MESH heading of publications: (Aborigin* or Indigen* or Torres Strait Island* or oceanic ancestry group or australoid*) and (wellbeing or health) and (Australia) and (child or maternal or parent* or women* or pregnan* or infan*) and (program* or service*) (n = 3507). Second, to maximise coverage of the grey literature, the same librarian searched 9 websites and clearinghouses related to Aboriginal and Torres Strait Islander child and maternal health. Included were the Australian Indigenous Health Infonet, Lowitja Institute, National Aboriginal Community Controlled Health Organisation, National Aboriginal and Torres Strait Islander Child and Maternal Health Exemplar Site Initiative, Telethon Institute for Child Health Research, Secretariat of National Aboriginal and Islander Child Care Resource Clearinghouse, Australian Institute of Aboriginal and Torres Strait Islander Studies, Australian Government Office of Aboriginal and Torres Strait Islander Health, and the Australian Institute of Family Studies (n = 1246). The date last searched was 17/05/2013. Third, a researcher (CJ) hand-searched the reference lists of reviews of Aboriginal and Torres Strait Islander health and wellbeing interventions identified by the electronic database search for relevant studies not yet identified (n = 12). The initial search yielded 4765 search results.

### Step 1: Identification of studies for exclusion

Studies were excluded if they: (a) were duplicates (n = 42); (b) did not focus on child and/or maternal health, or if the outcomes or predictor variables did not include or specifically relate to child and/or maternal health (n = 837); (c) did not focus on Indigenous people in Australia (n = 1587); (d) were not on primary health care programs and services (n = 429); e) were not journal articles, reports or book chapters (n = 621); f) were reviews, discussion papers, commentaries or case reports (n = 984); g) were focused only on child health (n = 8) and h) were published pre 1993 (n = 234). Step 1 excluded 4726 references, leaving 23 search results.

### Step 2: Classification of studies

The remaining 23 studies were examined to identify studies that were; 1) ***Intervention research:*** defined as studies which test the effectiveness of public health Aboriginal and Torres Strait Islander child and maternal health responses or examines the impact of interventions designed to alter health-related knowledge, attitudes or behaviours, or to improve health care delivery; or 2) ***Program descriptions:*** defined as literature which describes the methods or processes applied to implement a child and maternal health response***,*** but in which no data-based evaluation was reported [6].

### Data extraction

The characteristics of studies of Aboriginal and Torres Strait Islander child and maternal health responses were categorised by: 1) 1st author & year; 2) publication type and study type; 3) location and organisational setting; 4) intervention issue; 5) intervention types and components; 6) target group, sample; 7) outcomes or effects; and 8) study design and study quality (intervention studies only). Two researchers reviewed the publication characteristics and agreed on 22 out of 23, achieving 95.6% inter-rater reliability.

### Study quality assessment

Methodological quality of quantitative studies were assessed using the Dictionary for Effective Public Health Practice Project (EPHPP) Quality Assessment Tool for Quantitative Studies [13]. Sections A to F (A. selection bias; B. study design; C. confounders; D. blinding; E. data collection methods; and F. withdrawal and drop-outs) were coded weak, moderate or strong, consistent with the component rating scale of the Dictionary. For Sections G (intervention integrity) and H (analyses) descriptive information will be recorded, in line with the Dictionary recommendations. For qualitative studies,the Critical Appraisal Skills Program (CASP) quality assessment tool was used [14]. This tool assesses the clarity of study objectives, the quality of the methodology, research design, data collection and analyses, ethical considerations, whether there is a clear statement of findings and the value of the research. To assess the study quality of those using mixed-methods study design, the qualitative and quantitative components were assessed separately using both of the aforementioned tools.

## Results

Table 1 summarises the characteristics of included studies.

**Table 1:** **Aboriginal and Torres Strait Islander MIH program study characteristics**

1st author and year	Program/service name	Publication type and Study Type	Location and Organisation Setting	Intervention Issue	Intervention Type and Components	Target Group, and Sample Size	Outcomes or Effects	Study Design and Study Quality (intervention studies only)
Murphey, E. et al. (2012) [27]	The Aboriginal Maternal and Infant Health Service (AMIHS)	Journal Article Program description (Outcomes based on previous evaluation of the NSW Aboriginal Maternal and Infant Health Strategy)	NSW State wide Government strategy delivered through local government areas (LGA) with care provided in the community	Health of Aboriginal women during pregnancy and perinatal morbidity and mortality of Aboriginal infants	-Antenatal and postnatal care-Training and support for midwives and Aboriginal Health Workers	*Target Group:* Aboriginal women and their babies, from conception up to 8 weeks postpartum	-Increased antenatal attendance -Higher birth weights -Decreased preterm births -Decreased perinatal mortality -Improved breastfeeding rates	N/A – Program Description
NSW Health (2005) [18]	NSW Aboriginal Maternal and Infant Health Strategy (AMIHS)	Evaluation Report Intervention Research	NSW State wide Government strategy delivered through local government areas (LGA) with care provided in the community	Health of Aboriginal women during pregnancy and perinatal morbidity and mortality of Aboriginal infants	Targeted antenatal/postnatal programs	*Target Group:* Aboriginal women and their babies, from conception up to 8 weeks postpartum *Sample Size:* care provided to women in 2003 and 2004 n=689 people interviewed n=201	-Improved antenatal attendance prior to 20 weeks gestation-Improved breastfeeding rates -Decrease in low birth weight babies Decrease in perinatal deaths -No change in proportion of women smoking during pregnancy	*Study Design:* Prospective program specific quantitative data compared with population-based data from the NSW Midwives Data Collection. Qualitative data collected through Interviews and focus groups. *Study Quality:* Mixed-method Quantitative - Weak Qualitative - Weak
Boles, C. et al. (2005) [19]	The Alternative Birthing Project: Anangu Bibi	Conference paper Program description	Port Augusta and Whyalla SA – Northern and Far Northern Regional Health Service SA (rural) Delivered in people’s homes and across a range of local services and organisations including local Aboriginal Health Services.	Poor Aboriginal maternal and infant health, low antenatal attendance, low infant birth weight, high teenage pregnancy rates	-Continuum-of-care model led by Aboriginal maternal and infant care workers supported by midwives, GP and obstetrician Pregnancy checkups -Assessing for risk factors and education about early warning signs of complications-Health promotion and education-Postnatal support up to 8 weeks after birth	*Target Group:* Teenage mothers and young Aboriginal women	N/A	N/A – Program Description
Carter, E. et al. (2004) [20]	Congress Alukurra	Journal Article Intervention Research	Alice Springs, Central Australia (remote) ACCHO – Congress Alukurra	Women’s health	-Comprehensive antenatal and postnatal care-Shared maternity care -Gynaecological services -Sexual assault and domestic violence counselling and examinations -Health education -Transportation -Health worker training -Mobile bush clinic	*Target Group:* Aboriginal women and children *Sample Size:* Not available	-Increased client visits -Increase in women having first trimester antenatal visit -Slight increase in mean birth weight of infants from 1986-89 to 1991-95 and 1996-99.	*Study Design:* Analysis of documents and reports and secondary analysis of published and unpublished epidemiological data from Alukurra and the Northern Territory Midwives Collection. Routinely collected client information and changes in those factors over the previous five years was summarised. Interviews and consultations were conducted for qualitative study components. *Study Quality:* Mixed-method; Quantitative - Weak Qualitative – Moderate
Jan, S. et al. (2004) [21]	Daruk Aboriginal Medical Service Midwifery Program	Journal Article Intervention research	Mt Druitt, Western Sydney (urban) ACCHO – Daruk Aboriginal Medical Service	Aboriginal perinatal and maternal health	-Antenatal and postnatal care -Antenatal checkups -Hospital booking -Transport -Home visits -Labour support and delivery -Hospital visits -Assistance with infant feeding	*Target Group:* Aboriginal women/mothers and their infants *Sample Size:* Not available	-Lower gestational age at first visit -Higher number of antenatal visits -Women reported positive experiences with Daruk	*Study Design:* Measures of antenatal attendance and perinatal outcomes in clinical records were compared with the NSW Midwives Data Collection. Interviews and focus groups were used for the qualitative component. *Study Quality:* Mixed-method; Holistic economic evaluation Quantitative - Moderate Qualitative – Moderate
Australian Indigenous Health Infonet	Healthy for Life Maternal and Child Health Program, Derbarl Yerrigan Health Service	Webpage Program Description	Perth, WA (urban) ACCHO – Derbarl Yerrigan Health Service	Aboriginal maternal and child health	Goals: To improve pre-pregnancy health of women, increase pre-pregnancy immunisation, parental education and support, and home-visits	*Target Group:* Aboriginal women/mothers and children up to 5 years	N/A	N/A – Program Description
Campbell, S. et al. (2004)	Women’s Business Service Mildura	Journal Article Intervention research	Mildura, VIC (rural) ACCHO – Mildura Aboriginal Health Service	Maternity care	-Pregnancy screening -Antenatal and postnatal care -Education and information -Support during labour and birth -Check-up’s	*Target Group:* Aboriginal women in Mildura *Sample Size:* clients interviewed n=25 survey participants n=333	Women attending the service were significantly more positive about many aspects of their care than women attending other rural public maternity services	*Study Design:* Interviews were conducted using and structured interview schedule based on the Victorian Survey of Recent Mothers 2000. The views and experiences of women attending the Women’s Business Service were assessed and contrasted with those of rural women who participated in the 2000 state wide survey. *Study Quality:* Qualitative - Strong
Australian Indigenous Health Infonet	Moort Boodjari Mia (Family Pregnancy House)	Webpage Program description	Perth, WA (urban) Mainstream community health service – North Metropolitan Health Service	Maternal health care	-Antenatal and postnatal clinical care -Guidance, support and education	*Target Group:* Indigenous women, mothers and families, during pregnancy up to 4 weeks postpartum	N/A	N/A – Program Description
Panaretto, K. S. et al. (2007) [23]	Mums and Babies (MB) Program	Journal Article Intervention Research	Townsville, QLD (rural) ACCHO – Townsville Aboriginal and Islander Health Service	Aboriginal and Torres Strait Islander Infant and maternal health	-Integrated model of antenatal shared care Integrated team approach between Aboriginal Health Worker, midwives/child health nurses, Doctors and Obstetric team -Indigenous outreach health worker -Pregnancy registrar -Walk in clinic -Family orientation -Care plans -PCR testing for STI’s -Transport service Brief intervention for risk factors (smoking cessation, nutrition, antenatal education, breast feeding, sudden infant death syndrome)	*Target Group:* Indigenous women *Sample Size:* MB group n=781 PreMB group n=84	-Decrease in perinatal mortality -Increase in antenatal visits -Improvements in care planning -Completion of cycle-of-care -Antenatal education	*Study Design:* Comparative study with historical control group *Study Quality:* Quantitative - Weak
Panaretto, K. et al. (2005) [24]	Mums and Babies (MB) Program	Journal Article Intervention Research	Townsville, QLD (rural) ACCHO – Townsville Aboriginal and Islander Health Service	Aboriginal and Torres Strait Islander Infant and maternal health	Integrated model of antenatal shared care	*Target Group:* Indigenous women *Sample Size:* MB group n=45 PreMB group n=84 Contemporary control group n=540	-Increase in number of women attending the program who gave birth at hospital -Increase in number of antenatal visits -Reduction in pre-term births	*Study Design:* Comparative study with historical control group and contemporary control group *Study Quality:* Quantitative - Weak
Australian Indigenous Health Infonet	Nganampa Health Council Child and Maternal Health Program	Web page Program description	Anangu Pitjantjatjara/ Yankinytjatjaralands, SA (remote) ACCHO - Nganampa Health Council	Aboriginal child and maternal health	-An antenatal care program -Development and delivery of key messages health education packages for young mothers -Child health program: Protocolised growth monitoring for children under 5 years of age and targeted child health screening at ages 5, 10 and 14	*Target Group:* Aboriginal mothers and children 0-14 years	N/A	N/A – Program Description
Power, C. et al. (2008) [28]	Ngangkitta Ngartotdli Karpandi (Supporting Mums and Babies) Program	Evaluation Report Intervention Research	Adelaide, SA (urban) Joint government and community initiative	Aboriginal and Torres Strait Islander women and babies health	Framework for an integrated maternity care service for Indigenous women and their babies. Accessible and provides culturally responsive and timely maternity services	*Target Group:* Indigenous mothers and their babies *Sample Size:* Women enrolled in project during evaluation n=14	-All attending women had a antenatal plan -Women successfully engaged with the service -All women birthed at their local maternity service -Increase in referrals to appropriate support services -Women reported positive experiences of services	*Study Design:* Six phase Action Research design. Quantitative data collected from tools developed to measure key performance indicators were collated and compared to data for Aboriginal women and infants in the Southern Adelaide Health Service catchment area from the Pregnancy Outcomes Unit of the South Australian Department of Health. Face to face interviews with clients and telephone interviews with service providers were conducted for the qualitative study component. *Study Quality:* Mixed-method; Action Research - Quantitative - Weak Qualitative - Strong
Dorman, R. et al. (1997) [15]	Ngua Gundi (Mother and Child) Program	Journal Article Program Description	Rockhampton, QLD (rural) Mainstream community health service	Indigenous maternal and infant health	-Antenatal clinic -Midwifery model of care - referrals to other medical practitioners -home visits -antenatal education -under 5’s clinic	*Target Group:* Indigenous mothers and children from pre-pregnancy to 5 years	N/A	N/A – Program Description
Smith, R. M. et al. (2000) [16]	Strong Women, Strong Babies, Strong Culture (WA)	Journal Article Intervention Research	Aboriginal communities across the Kimberley and Pilbara regions, WA (remote) Community initiated program delivered by Aboriginal women across 5 communities. SW,SB,SC is a government developed program administered through the Territory Health Services Darwin	Infant birth weights and child growth	-Nutritional intervention to improve birth weights and growth of infants and children -Nutritional assessment of infants and children -Counselling of mothers and carers -Implementation of maternal support program	*Target Group:* Aboriginal mothers and children 0-3 years *Sample Size:* Not available	The intervention was not accompanied by any change in full-term birth weight but was associated with increased weight gain after 6 months.	*Study Design:* Comparative study with historical control group *Study Quality:* Quantitative - Weak
Mackerras, D. (2001) [17]	Strong Women, Strong Babies, Strong Culture (NT)	Journal Article Intervention Research	NT (remote) Community-based program – program developed by the Northern Territory Department of Health and Community Services in conjunction with Aboriginal people	Infant birth weight	-Increased attendance for antenatal care in first trimester -Risk assessment for potential complications -Introduced nutritional assessment and monitoring into prenatal care -Strategies to improve maternal nutrition and increase maternal weight gain	*Target Group:* Aboriginal women who are pregnant or of childbearing age *Sample Size:* Not available	-Increase in the mean birth weight of infants of Aboriginal women -Changes in maternal weight were associated with changes in birth weight over time	*Study Design:* Data from the NT Midwives Collection and from antenatal charts for births in the communities were used to determine changes in mean birth weights of infants in the three pilot communities compares to other NT communities. *Study Quality:* Quantitative - Weak
Tursan d’Espaignet, E. et al. (2003) [25]	Strong Women, Strong Babies, Strong Culture (NT)	Journal Article Intervention research	NT (remote) Community-based program – program developed by the Northern Territory Department of Health and Community Services in conjunction with Aboriginal people	Perinatal health and infant birth weight	-Senior women in communities helping younger women prepare for pregnancy -Antenatal care -Advice and encouragement to improve nutrition (including increase in use of bush foods) -Encouraging reduction in alcohol and tobacco consumption Encouragement to seek medical assistance	*Target Group:* Aboriginal women who are pregnant or of childbearing age *Sample Size:* Group 1 Pre-intervention n=577 Post-intervention n=829 Control group 1 Pre n=2118 Post n=3070 Group 2 Pre n=814 Post n=322 Control group 2 Pre n=3511 Post n=1677	-Significant improvements in infant birth weight was reported in one intervention group	*Study Design:* A comparison of pre and post intervention birth weights in intervention and control communities was performed *Study Quality:* Quantitative - Weak
Crook, L. et al. (2012) [29]	Waminda Mums and Bubs Program	Journal Article Program description	Nowra, NSW South Coast (rural) ACCHO – Women’s health and welfare service	Aboriginal maternal and child health	-Antenatal and postnatal care -Health and development information about infant care -Practical advice and assistance with breastfeeding, nutrition and parenting skills -Monitoring children’s weight, immunisation status and growth milestones -Early testing to detect issues with children’s hearing, sight speech and other developmental issues prior to commencing school - Health checks -Physical examinations -Screening -Pathology -Home visiting -Immunisation -Health assessments -Education sessions	*Target Group:* From pre-conception, to antenatal, birthing, postnatal and continuous care (lifelong care for all females and males up to 14 years)	N/A	N/A – Program Description
Adams, E. et al. (2011) [30]	Winnunga Nimmityjah Perinatal and infant mental health service	Journal Article Program description	ACT (urban) ACCHO - Winnunga Nimmityjah	Perinatal and infant social and emotional wellbeing (mental health)	Perinatal and infant mental health service	*Target Group:* Aboriginal mothers and their babies	N/A	N/A – Program Description
Wong, R. et al. (2011) [31]	Winnunga Nimmityjah Aboriginal Midwifery Access Program (AMAP)	Journal Article Intervention Research	ACT (urban) ACCHO - Winnunga Nimmityjah	Aboriginal maternal and child health	-Antenatal care -Birth support -Postnatal care - Full antenatal care -Home visits -Assistance with appointments -Transport -Birth support -Post natal follow up -immunisations	*Target Group:* Aboriginal and Torres Strait Islander women in ACT *Sample Size:* Attended AMAP Women n=187 Babies n=193	AMAP clients had -Higher smoking rates -Lower caesarean ate -Lower proportion of pre-term births -Lower proportion of low birth weight babies	*Study Design:* Comparison between AMAP client data and ACT Maternal and Perinatal Collection data *Study Quality:* Quantitative - Weak
Australian Indigenous Health Infonet	Wurli Wurlinjang child and maternal/ women’s health program	Web page Program description	Katherine and surrounding areas, NT (remote) ACCHO - Wurli Wurlinjang	Child and maternal/women’s health	-Health promotion and education. Preventative health care -childhood immunisations -growth assessment -child health checks -education around substance misuse and nutrition in early childhood development PAP screening	N/A	N/A	N/A – Program Description
Australian Indigenous Health Infonet	Boodjari Yorda (Pregnant women’s) Program	Web page Program description (Outcomes provided with no supporting documentation)	Wheat belt region, WA (rural) Government program	Women’s/maternal health	-Antenatal and postnatal care -Home visits -Assistance attending appointments -Sexual and reproductive health education -Nutrition education support	*Target Group:* Indigenous women and their families	Reduction in overdue immunisations and improved nutritional status	N/A – Program Description
Australian Indigenous Health Infonet	Moorditj Boodjaree yorgers (maternal health)	Web page Program description	Bentley-Armadale area, WA (urban) Mainstream community service – Medicare Local	Maternal health	-Antenatal and postnatal support -Home check ups -Information, education and resources about pregnancy, nutrition and taking baby home -At home post-natal support up to 6 weeks after birth -Information and resources about breastfeeding, immunisation, services and groups	*Target Group:* Indigenous mothers and babies up to 6 weeks postpartum	N/A	N/A – Program Description
Office for Aboriginal and Torres Strait Islander Health (OATSI H), (2005) [26]	Aboriginal and Torres Strait Islander Child and Maternal Health Exemplar Site Initiative: Sire Reports 2005 Nganampa Health Council Child and Maternal Health Program (SA) Townsville Mums and Babies Program (QLD) Durri Aboriginal Medical Service Djuli Galban Program (NSW)	Report Intervention Research	Anangu Pitjantjatjara/ Yankinytjatjara lands, SA , Townsville QLD, Kempsey NSW (remote and rural) ACCHO - Nganampa Health Council ACCHO - Townsville Mums and Babies Program Aboriginal Medical Service - Durri Aboriginal Medical Service	Indigenous maternal and child health	*Nganampa* Antenatal care program -Development and delivery of Key Messages Health Education Packages for young mothers -Child health program including; childhood immunisation, protocolised growth monitoring for under 5’s, and targeted health checks at ages 5, 10 and 14 *Mums and Babies* -maternal/paternal and child health, acute care, preventive care and follow-up -one-on-one education/health promotion (eg antenatal and postnatal health, nutrition, substance use, family violence) -transport -shared antenatal care with the Townsville Hospital -immunisation -growth and developmental monitoring -Referral, advocacy and social support. *Djuli Galban* -Antenatal and postnatal services -Brief intervention education around risk factors such as smoking and drug use during pregnancy -Child health services -Immunisation services	*Target Group:* Indigenous women/mothers and children *Sample Size:* Not available	*Nganampa* Earlier antenatal attendance Increased antenatal attendance -Decrease in babies born with low birth weight -Decreased rates of malnutrition and stunting -Higher rates of childhood immunisation coverage *Mums and Babies* -Increased access to antenatal care -Increase in number of antenatal births per pregnancy -Decrease in pre-term births -Decrease in babies born with low birth weight -Increase in mean birth weight *Djuli Galban* -High rates of antenatal attendance prior to 20 weeks gestation -No significant changes in rates of pre-term births and babies with low birth-weight -Increased rates of childhood immunisation	Information on study design and methods not provided Weak

### Publication year and study design

From a low during the 1990’s, there was a significant increase in publications from 2003. With only three studies published in the period from 1993–2002 (13%) [15–17], the largest number of publications found were for the period 2003–2007 (9/23, 39%) [18–26] and another five (22%) were published in the period 2008-2012 [27–31]. Publication dates were not available for a further 6 (26%) search results as these were from websites and no dates were recorded [32–37]. Fifty two percent (12/23) of the publications were intervention studies i.e., evaluations of programs or services [16–18, 20–26, 28, 31]. The other 48% (11/23) were program descriptions [15, 19, 27, 29, 30, 32–37].

### Location and organisation setting

The programs and services identified in the literature operated from a range of locations throughout Australia. The distribution of documented programs and services by state was Western Australia (5/23, 22%) [16, 32–34, 37], New South Wales (4/23, 17%) [18, 21, 27, 29], the Northern Territory (4/23, 17%) [17, 20, 25, 36], Queensland (3/23, 13%) [15, 23, 24], South Australia (3/23, 13%) [19, 28, 35], the Australian Capital Territory (2/23, 9%) [30, 31] and Victoria (1/23, 4%) [22]. One additional publication reported on three programs operating in South Australia, New South Wales and Queensland [26].

The majority of publications reported on programs and services operating from Aboriginal Community Controlled Health Organisations (ACCHO) (12/23, 52%) [20–24, 26, 29–31, 35–37]. There were 5 publications documenting government programs (22%) [17, 18, 25, 27, 32] and 3 documenting programs operating out of mainstream primary health services (13%) [15, 33, 34]. One study documented a program delivered by an Aboriginal Maternal and Infant Care team across a range of sites, including local Aboriginal Health Service’s [19]. Another study was on a community initiated program operating across several communities using a government developed program delivered by local Aboriginal women [16]. There was also an evaluation of a joint government and community initiative [28].

### Target population and intervention issue

Six of the publications stated the target group for programs and services generally as Aboriginal and Torres Strait Islander mothers and their children/babies/families (6/23, 26%) [20, 21, 26, 28, 30, 32] without specifying whether care was provided pre-pregnancy and until what age after birth. Aboriginal and/or Torres Strait Islander or Indigenous women (4/23, 17%) [22–24, 31], Aboriginal women who are pregnant or of childbearing age [17, 25] and teenage mothers and young Aboriginal mothers [19] were also stated target groups. Several of the studies documented programs and services which provide post natal and child health services for infants and children of ages varying from 4 weeks up to 14 years [24–31]. There were only three publications which specified that the service was for women prior to pregnancy [15, 20, 29].

Nineteen of studies reviewed (19/23, 83%) [15, 18–24, 26–29, 31–33, 36, 37] identified a general intervention issue (eg. ‘perinatal and maternal health’, ‘maternity care’ and ‘child and maternal health’) which were grouped under the broad category of Aboriginal and Torres Strait Islander maternal and child health and wellbeing. This general categorisation is underpinned by an extensive literature base outlining the key MCH needs of Aboriginal and Torres Strait Islander peoples targeted by programs and services. More specific or targeted intervention issues such as low antenatal attendance [19], low infant birth weight [16, 17, 19, 25], high teenage pregnancy rates [19], poor child growth [16] and perinatal and infant social and emotional wellbeing (mental health) [30] were also referred to in the literature.

### Intervention type and components

Antenatal and postnatal care were identified as the main intervention types in 14 (61%) of the search documents [15, 17, 18, 20–22, 25, 27, 29, 31–33]. Another 4 (17%) publications identified an integrated or continuum model of maternity care as the main intervention [19, 23, 24, 28]. One publication documenting a service targeting mothers and infants identified health promotion and education as the primary intervention [36], another identified advocacy, support and psychotherapy as the main interventions [30] and two did not state a main intervention type [16, 37].

The most common component of interventions cited in the literature documenting MCH was health promotion/education and advice/support (16/23, 70%) [15, 16, 19, 20, 22, 23, 25, 26, 29, 30, 32, 33, 36, 37]. Health promotion topics documented included nutrition [23, 25, 32, 33, 36], breastfeeding [23, 33], immunisation [33], infant care [33] and accessing groups and services [33]. Publications that described or evaluated these health promotion/education and advice/support activities focused on the health issues of smoking cessation [23], sexual and reproductive health [32], substance misuse [25, 36], early warning signs of complications [19] and Sudden Infant Death Syndrome (SIDS) [23]. Other common intervention components documented in the literature include home visitation (8/23, 35%) [15, 26, 29, 31–33, 36, 37], antenatal and postnatal checkups and support (5/23, 22%) [19, 22, 31, 33, 36], transport services (4/23, 17%) [20, 23, 31, 36], labor/birth support (3/23, 13%) [22, 31, 36], assistance making or attending appointments and hospital bookings (3/23, 13%) [21, 31, 32], pregnancy screening (2/23, 9%) [22, 29], counseling/psychotherapy (2/23, 9%) [16, 30], referrals (2/23, 9%) [15, 26] and training and support for midwives and Aboriginal Health Workers (2/23, 9%) [20, 27].

### Outcomes and effects

Of the 23 publications, 14(61%) reported program outcomes and/or effects. These included the 12 intervention studies plus two program descriptions (one was based on an evaluation report [27] and the other simply claimed outcomes without providing evidence [32]). The outcomes and effects described in the literature on maternal and infant health services included an increase in antenatal attendance (6/23, 26%) [20, 21, 23, 24, 26, 27], an increase in infant birth weights (5/23, 22%) [17, 20, 25–27], a decrease in, or lower proportion of, pre-term births (4/23, 17%) [24, 26, 27, 31], earlier antenatal attendance (4/23, 17%) [18, 20, 21, 26] and a decrease in, or lower proportion of, low birth weight babies [18, 26, 31]. Decreased perinatal mortality [18, 23, 27], reports of positive views and/or experiences of the service from service users [21, 22], improved breastfeeding rates [18, 27] and improved nutritional status such as decreased rates of stunting and malnutrition [26, 32] were also reported. Other outcomes included higher rates of childhood immunisation coverage, improvements in care planning [23], lower caesarean rates [31] and changes in birth weight associated with changes in maternal weight [17]. Completion of cycle of care [23] and reduction in overdue immunisations [32] were each cited as outcomes along with an increase in infant weight after six months [16]. One study reported the outcomes of all women having an antenatal plan, women having successfully engaged with the service, all women having birthed at their local maternity service and an increase in referrals to appropriate support services [28]. One evaluation study described no changes in the proportion of women smoking during pregnancy [18].

### Methodological quality of intervention studies

The 12 intervention studies were assessed for study quality. All 6 studies which employed solely quantitative methods were rated as weak. There were four mixed-method studies, three of which were rated as weak for the quantitative aspect, and the other moderate. Quantitative studies most commonly received weak ratings for confounders, data collection methods and withdrawals and drop outs (see Tables 2 and 3 for further details on study quality assessments). For the qualitative component of mixed-method evaluations, two were rated moderate, one weak, and the other strong. There was a further qualitative study which was rated strong, and one study that was rated weak due to lack of information regarding the study design and methods used.

**Table 2:** **Effective Public Health Practice Project (EPHPP) quality assessment of quantitative studies**

Publication	Selection bias	Study design	Confounders	Blinding	Data collection methods	Withdrawals and dropouts	Intervention integrity*	Analyses**	Total score
Jan, S., et. al. (2004) [21]	Moderate	Moderate	Weak	Moderate	Weak	Not applicable	(Q1) Can’t tell	(Q1) Individual	Moderate
							(Q2) Can’t tell	(Q2) Organisation	
							(Q3) Can’t tell		
								(Q3) Yes	
								(Q4) No	
Carter, E. et al. (2004) [20]	Moderate	Moderate	Weak	Weak	Weak	Not applicable	(Q1) Can’t tell	(Q1) Individual	Weak
							(Q2) Can’t tell	(Q2) Organisation	
							(Q3) Can’t tell		
								(Q3) Yes	
								(Q4) No	
NSW Health (2005) [18]	Moderate	Moderate	Weak	Weak	Weak	Weak	(Q1) 80-100%	(Q1) Individual	Weak
							(Q2) Can’t tell	(Q2) Individual	
							(Q3) Can’t tell		
								(Q3) Yes	
								(Q4) No	
Power, C. et al. (2008) [28]	Weak	Weak	Weak	Weak	Weak	Weak	(Q1) 80-100%	(Q1) Individual	Weak
							(Q2) Can’t tell	(Q2) Individual	
							(Q3) Can’t tell	(Q3) Yes	
								(Q4) No	
Panaretto, K. S. et al. (2007) [23]	Moderate	Moderate	Weak	Moderate	Weak	Weak	(Q1) 80-100%	(Q1) Individual	Weak
							(Q2) No	(Q2) Individual	
							(Q3) Can’t tell		
								(Q3) Yes	
								(Q4) No	
Panaretto, K. et al. (2005) [24]	Moderate	Moderate	Weak	Moderate	Weak	Weak	(Q1) 80-100%	(Q1) Individual	Weak
							(Q2) No	(Q2) Individual	
								(Q3) Yes	
							(Q3) Can’t tell		
								(Q4) No	
Smith, R. M. et al. (2000) [16]	Moderate	Moderate	Weak	Moderate	Weak	Weak	(Q1) 80-100% (Q2) Can’t tell	(Q1) Community	Weak
							(Q3) Can’t tell		
								(Q2) Individual	
								(Q3) Yes	
								(Q4) No	
Mackerras, D. (2001) [17]	Moderate	Moderate	Weak	Moderate	Weak	Weak	(Q1) Can’t tell	(Q1) Community	Weak
							(Q2) No		
							(Q3) Can’t tell	(Q2) Individual	
								(Q3) Yes	
								(Q4) No	
Tursan d’Espaignet, E. et al. (2003) [25]	Moderate	Moderate	Weak	Moderate	Weak	Weak	(Q1) Can’t tell	(Q1) Community	Weak
							(Q2) Can’t tell		
							(Q3) Can’t tell	(Q2) Individual	
								(Q3) Yes	
								(Q4) No	
Wong, R. et al. (2011) [31]	Moderate	Moderate	Weak	Moderate	Weak	Weak	(Q1) Can’t tell	(Q1) Individual	Weak
							(Q2) Can’t tell	(Q2) Individual	
							(Q3) Can’t tell		
								(Q3) Can’t tell	
								(Q4) No	

*Intervention Integrity.

Q1) What percentage of participants received the allocated intervention or exposure of interest.

Q2) Was the consistency of the intervention measured?

Q3) Is it likely that participants received an unintended intervention (contamination or co-intervention) that may influence the result?

**Analyses.

Q1) Unit of allocation.

Q2) Unit of analyses.

Q3) Are the statistical methods appropriate for the study design?

Q4) Is the analyses performed by intervention allocation status (i.e. intention to treat) rather than the actual intervention received?

**Table 3:** **Critical Appraisal Skills Program (CASP) quality assessment of qualitative studies**

Publication	Clear statement of research?	Qualitative methodology appropriate?	Research design appropriate for aims?	Recruitment strategy appropriate for aims?	Data collection addresses research issue?	Relationship between researcher and participant considered?	Ethical considerations accounted for?	Rigorous data analysis?	Clear statement of findings?	Research is valuable?	Total score
Jan, S., et. al. (2004) [21]	Yes	Yes	Can't tell	Yes	Yes	No	No	Yes	Yes	Yes	Moderate
Carter, E. et al. (2004) [20]	Yes	Yes	Yes	Yes	Yes	Yes	Can't tell	Yes	Yes	Can't tell	Moderate
Campbell, S. et al. (2004)	Yes	Yes	Yes	Yes	Yes	Yes	Yes	Yes	Yes	Yes	Strong
NSW Health (2005) [18]	Yes	Yes	No	No	No	No	Yes	No	Yes	Yes	Weak
Power, C. et al. (2008) [28]	Yes	Yes	Yes	Yes	Yes	No	Yes	Yes	Yes	Yes	Strong

## Discussion

This systematic literature review contributes detailed information about the characteristics of programs and services for Aboriginal and Torres Strait Islander MCH. Results pertaining to the reported outcomes or effects and the lack of high quality evaluations mirror the findings of previous reviews of Aboriginal and Torres Strait Islander MCH programs and services. This paper extends the existing knowledge base by updating the literature search and contributing new findings about the organisational settings, program content and components, and reported outcomes of MCH programs and services. As well, our review of study quality draws attention to the gaps in evidence. Building on the knowledge presented in previous reviews, the conclusions drawn here contribute to the broader understanding of what is required to improve the health and wellbeing of Aboriginal and Torres Strait Islander mothers and infants.

### Organisation setting

The finding that over half of publications reviewed (52%) reported on MCH programs operating out of ACCHO’s is significant considering the connection between the need for self-determination and Aboriginal and Torres Strait Islander health and wellbeing. A lack of power and control has long been recognised as contributing to the health inequality experienced by Aboriginal and Torres Strait Islander peoples, hence the significance and continuing use of community control of decision-making processes and resources as a health improvement strategy [38]. Along with the prevalence of community controlled MCH care programs, a further two studies reviewed documented a community-based program developed by the NT government in conjunction with Aboriginal people [17, 25] and another one on a community initiated program developed and administered by the government [16]. These findings supports that of Herceg [7] which found that being community based and/or community controlled is one factor important in successful Aboriginal and Torres Strait Islander MCH programs around Australia. This strong presence of community controlled and community-based or initiated programs is a positive indicator of efforts to enhance the overall wellbeing, not only of Aboriginal and Torres Strait Islander women and their babies, but also of the communities in which they live. It is important to note, however, that 22% of publications being on government programs and services and 13% on programs operating out of mainstream community health services demonstrates the important role that the mainstream primary health sector has an to play in addressing the health needs of Aboriginal and Torres Strait Islander mothers and infants.

### Program content and components

In regards to the types of responses outlined, the main intervention type reported in the publications was antenatal and postnatal care (61%), however details of what this care entailed were not provided. There are currently no Australian national guidelines for the provision of antenatal care and research has found that, although protocols used in different antenatal care settings have common areas, there is significant variation in coverage and recommendations about schedules and tests [39]. In the absence of thorough documentation of tests, screening procedures and treatments undertaken in the studies reviewed, it was impossible to assess quality of antenatal and postnatal care for Aboriginal and Torres Strait Islander women and infants. A further 17% of publications identified an integrated or continuum of care model as the main intervention type. A lack of continuity of care has been identified as a common issue affecting communication and quality of care in antenatal and postnatal services for Aboriginal and Torres Strait Islander women [39]. Research shows that fragmented maternity care can increase medical risks and compromise patient safety, causing adverse outcomes for women and infants [40–42]. This focus on models of continuity of care demonstrates the efforts of several Aboriginal and Torres Strait Islander MCH programs to address these concerns and ensure quality care that meets the needs of the women using these services.

The most common component of MCH interventions, documented in 70% of the literature reviewed, was health promotion/education and advice/support, a finding not identified in previous reviews. Education and health promotion is a prominent MCH care strategy, particularly used by midwives who emphasise their role in promoting the health and wellbeing of pregnant women [43]. Brief health education interventions have been shown to reduce alcohol [44] and tobacco [45] consumption during pregnancy and nutritional education and counseling has been associated with improved gestational weight gain, a reduced risk of anaemia, an increase in infant birth weight and a decreased risk of pre-term birth [46]. Research has found that the provision of additional support for at-risk pregnant women reduced the likelihood of caesarean births and hospital admissions, however no impact was shown on the incidence of babies born with low birth weight, pre-term birth, perinatal deaths, maternal satisfaction with care or depression [47]. While there appears to be a lack of evidence documenting the effectiveness of health education/promotion and advice/support for pregnant Aboriginal and Torres Strait Islander women, it has been suggested that, embedded within a broader antenatal program, these approaches can contribute to better birth outcomes by improving cultural safety and engagement [48].

Home visitation and transport services were other intervention components commonly found in the literature, consistent with the findings of Herceg [7]. However, while there were common program components seen across multiple studies, more apparent was the diversity of responses in MCH programs. This may be a reflection of the focus of primary health care in Aboriginal and Torres Strait Islander contexts, which is on providing appropriate, holistic care to meet the unique health needs of each community [49]. However, this lack of consistency between Aboriginal and Torres Strait Islander MCH programs means it is difficult to assess the effects of particular components or combinations of components on the pregnancy and birth outcomes of mothers and infants. More rigorous evaluations which study the impact of specific program components would be needed to further explore this issue.

This review also identified some significant gaps in the literature. Consistent with previous research indicating the underutilisation of smoking cessation interventions with Aboriginal and Torres Strait Islander women who smoked during pregnancy [39], this review found documentation of interventions targeting smoking during pregnancy in only two publications reviewed [23, 25]. While there is evidence that smoking cessation interventions cannot only help women reduce or stop smoking but also have an impact on birth weight and pre-term birth [45], a recent systematic review found that there is currently no evidence for effective smoking cessation interventions for pregnant Aboriginal and Torres Strait Islander women [50]. Given the current lack of evidence, there is a need for high quality evaluations of approaches to smoking cessation among pregnant Aboriginal and Torres Strait Islander women [50].

Another important factor found to be lacking in evaluations was women’s subjective views and experiences of their health care, with only two publications reporting on this [21, 22]. The need to consider and prioritise the feelings, experiences and preferences of Aboriginal and Torres Strait Islander women regarding their pregnancy is consistently reiterated in the literature [39, 51, 52] and is something that needs to be addressed in future evaluations. There was also a lack of documentation on programs which linked women’s pre-conception health to pregnancy and birth outcomes. Only three publications explicitly stated that the service was available for non-pregnant women [17, 25, 29], with only one program stating goals to improve the pre-conception health of women and increase pre-conception immunisation [37]. This finding supports that of previous research which has shown that many women to do not receive pre-conception advice from health care professionals and that most women have low levels of knowledge about pre-conception health care, despite the strength of evidence on the positive benefits of this for MCH outcomes [53]. There is a strong imperative to enhance the pre-conception health of Aboriginal and Torres Strait Islander women given that many factors which contribute to the poor birth outcomes of Aboriginal and Torres Strait Islander are preventable, especially when addressed before pregnancy [54].

### Outcomes and study quality

Similar to those reported by Eades [8] and Herceg [7], many of the publications reviewed reported positive outcomes. The main outcome identified across the studies on MCH programs was an increase in antenatal attendance (26%), with earlier antenatal attendance also reported in several evaluations (17%). This is an important outcome considering the late and low antenatal attendance rates typical of Aboriginal and Torres Strait Islander women [9, 39] and concerns around poor management of complications resulting in increased morbidity and mortality [27]. However, while equity in antenatal care utilisation is important, it should not be assumed that increased antenatal coverage will necessarily impact on other maternal and infant health outcomes [55]. Hunt [39] demonstrated that when programs of enhanced antenatal attendance have been rigorously evaluated, controlling for confounding factors such as socio-economic status, earlier and more frequent antennal care did not improve perinatal outcomes, such as low birth weight [39]. Equally important to antenatal utilisation was a focus on aspects of quality care, such as ensuring that recommended brief interventions, advice, screening procedures and treatments were adhered to [56].

Other key outcomes reported includedan increase in infant birth weights after birth (22%) and a decrease in, or lower proportion of, infants born with low birth weight (13%). Although birth weight is a crucial measure of maternal and infant health being associated with a range of poor health outcomes throughout the lifetime [48, 57], it is also a measure that requires further investigation. While pregnancy is important in determining the birth weight of babies, there are many other influencing factors, such as socio-economic status, generational maternal nutrition, health risk behaviours, maternal age and size, and medical conditions during pregnancy [52, 55]. Not discounting the importance of these key indicators, it is necessary to critically examine the primary MCH measures used to assess the effectiveness of MCH programs for enhancing the health and wellbeing of Aboriginal and Torres Strait Islander women and infants.

The outcomes reported in the literature reviewed paint a positive picture for progress in improving the health outcomes of Aboriginal and Torres Strait Islander mothers and infants. However it is impossible to conclusively state any cause and effect relationships between these and MCH programs due to issues of study quality. The methodological quality of intervention studies varied considerably, with quantitative evaluations and the quantitative components of mixed-method studies overwhelmingly rated as weak. Cluster Randomised Control Trials are an appropriate design for evaluations of interventions that naturally occur in groups of individuals [58] and should be explored further for use in evaluations of primary health care interventions. Multiple Baseline research designs have also been recommended as an alternative to Randomised Control Trials for methodologically rigorous and practical evaluations of population-based health interventions [59]. However we found no studies that utilised these higher level designs. As found in previous reviews of Aboriginal and Torres Strait Islander MCH care programs, good quality longitudinal data on MCH care is needed to demonstrate clinically significant perinatal outcomes [9]. It is also important to consider that, given the disparities in health outcomes faced by Aboriginal and Torres Strait Islander women and children compared to non-Indigenous women and infants, significant improvements in MCH outcomes are unlikely to be achieved over a short period of time. For this reason, it is critical that a long term vision is taken for assessing the impact of antenatal care programs and services.

### Limitations

Although a rigorous and thorough search strategy was employed, limitations include the possibility that the search did not locate all relevant studies. There is also a risk for publication bias in that programs and services which resulted in no overall benefit, or harmful effects may not have been reported on or published, and would therefore would not be included in this search. Other programs and services are likely to exist but were not included in this review because there was no publicly available literature concerning them. Since evaluations with statistically significant findings are more likely to be published, it is also possible that the published evaluations reviewed may over-estimate the true effectiveness of interventions [60]. The general weakness of quantitative studies is consistent with previous reviews of Indigenous intervention research [12]. While this weakens the evidence base of reported outcomes, it also provides an opportunity for researchers to improve the quality of evaluations of Indigenous health and wellbeing programs and services through the application of more rigorous study designs.

## Conclusion

There has been a substantial increase in publications documenting Aboriginal and Torres Strait Islander child and maternal health programs and services over the past decade. These publications outline responses to a range of health and wellbeing issues relevant to Aboriginal and Torres Strait Islander mothers, infants and children, with the majority of studies documenting programs targeting mothers and their babies through antenatal and postnatal care. There was a significant difference in the components of programs and services outlined in the literature, with health promotion/education and advice/support reported across a majority of maternal and infant health publications. The literature documents a serious underutilisation of tobacco smoking cessation interventions in antenatal care. Some improvements in health outcomes were reported on, however the true effectiveness of interventions documented in the quantitative studies is unclear due to poor study quality. Considering that it is unlikely that significant improvements in health outcomes will be achieved in the short term, research efforts need to focus on developing good quality longitudinal data to assess the impact of Aboriginal and Torres Strait Islander child and maternal health programs and services over several decades.
